# Alterations in the brain interactome of the intrinsically disordered N-terminal domain of the cellular prion protein (PrP^C^) in Alzheimer’s disease

**DOI:** 10.1371/journal.pone.0197659

**Published:** 2018-05-23

**Authors:** Sarah Ulbrich, Petra Janning, Ralf Seidel, Jakob Matschke, Anika Gonsberg, Sebastian Jung, Markus Glatzel, Martin Engelhard, Konstanze F. Winklhofer, Jörg Tatzelt

**Affiliations:** 1 Department Biochemistry of Neurodegenerative Diseases, Institute of Biochemistry and Pathobiochemistry, Ruhr University Bochum, Germany; 2 Max Planck Institute of Molecular Physiology, Dortmund, Germany; 3 Institute of Neuropathology, University Medical Center Hamburg-Eppendorf, Hamburg, Germany; 4 Department Molecular Cell Biology, Institute of Biochemistry and Pathobiochemistry, Ruhr University Bochum, Germany; INRA Centre de Jouy-en-Josas, FRANCE

## Abstract

The cellular prion protein (PrP^C^) is implicated in neuroprotective signaling and neurotoxic pathways in both prion diseases and Alzheimer’s disease (AD). Specifically, the intrinsically disordered N-terminal domain (N-PrP) has been shown to interact with neurotoxic ligands, such as Aβ and Scrapie prion protein (PrP^Sc^), and to be crucial for the neuroprotective activity of PrP^C^. To gain further insight into cellular pathways tied to PrP, we analyzed the brain interactome of N-PrP. As a novel approach employing recombinantly expressed PrP and intein-mediated protein ligation, we used N-PrP covalently coupled to beads as a bait for affinity purification. N-PrP beads were incubated with human AD or control brain lysates. N-PrP binding partners were then identified by electrospray ionization tandem mass spectrometry (nano ESI-MS/MS). In addition to newly identified proteins we found many previously described PrP interactors, indicating a crucial role of the intrinsically disordered part of PrP in mediating protein interactions. Moreover, some interactors were found only in either non-AD or AD brain, suggesting aberrant PrP^C^ interactions in the pathogenesis of AD.

## Introduction

Prion diseases in humans and other mammals are characterized by a conformational transition of the cellular prion protein (PrP^C^) into an aberrantly folded isoform, designated Scrapie prion protein (PrP^Sc^). PrP^Sc^ can form amyloid plaques in the diseased brain and is the major constituent of infectious prions (rev. in [1–4]). In the absence of PrP^C^ mice are resistant to prion diseases and cannot propagate infectious prions [5]. Moreover, expression of PrP^C^ in neurons mediates neurotoxic effects of Scrapie-prions [6–9], amyloid beta (Aβ) [10–13], and α-synuclein [14].

Structural studies revealed that PrP^C^ is composed of two major domains of similar size. The structured C-terminal domain contains three alpha-helical regions and a short, two-stranded beta-sheet, while the N-terminal domain is intrinsically disordered [15–17]. Originally, the activity of a protein was thought to be linked to the ability of the polypeptide chain to adopt a stable secondary/tertiary structure. This concept was extended when it became evident that intrinsically disordered domains (IDDs) and proteins can participate in a broad range of defined physiological activities and play a major role in several protein classes. Specifically, IDDs can bind to different partner molecules with diverse functional consequences [18–21].

Most of the unstructured domain of PrP^C^ is dispensable for the formation of infectious prions and PrP^Sc^: PrP27-30, the protease K-resistant core of PrP^Sc^, lacks amino acids (aa) 23–90, but is still infectious [22, 23]. Similarly, propagation of infectious prions is supported in transgenic mice expressing only a truncated version of PrP devoid of aa 32–93 [24]. However, various approaches in transgenic mice and cultured cells including primary neurons revealed that the N-terminal domain (N-PrP) modulates the signaling activity of PrP^C^. Strikingly, this role of N-PrP is diverse and seemingly contradictory. For example, the physiological function of PrP^C^ to promote neuronal viability under various stress conditions is linked to this domain. On the other hand, the toxic activity of certain PrP mutants and the ability of PrP^C^ to mediate neurotoxic signaling of Aβ and PrP^Sc^ is also dependent on N-PrP (rev. in [12, 25–27]). In this context it is important to note that N-PrP is generated *in vivo* via the proteolytic processing of mature PrP^C^ by a yet unknown protease [28–31], indicating a specific function of the shedded domain. A soluble N-terminal fragment of PrP^C^ was described to promote peripheral myelination by activating the G protein-coupled receptor Adgrg6 [32]. Strikingly, the mode of action of N-PrP might change after it is released from PrP^C^. In the context of full-length GPI-anchored PrP^C^ the N-terminal domain mediates neurotoxic effects of Aβ while a secreted version abrogates Aβ-induced toxicity, presumably by trapping toxic Aβ species [13, 33–35]. To mechanistically understand these different properties of N-PrP, the identification of interacting proteins is important.

So far, PrP-interacting proteins were identified by using recombinant or cellular full-length PrP as a bait. Here, we present a novel approach to generate and employ N-PrP coupled to beads for the analysis of the brain interactome of PrP. Our study emphasizes the prominent role of the unstructured N-terminal domain in mediating PrP^C^-protein interactions and reveals alterations of the PrP interactome in Alzheimer’s disease.

## Results and discussion

**Fig 1:**
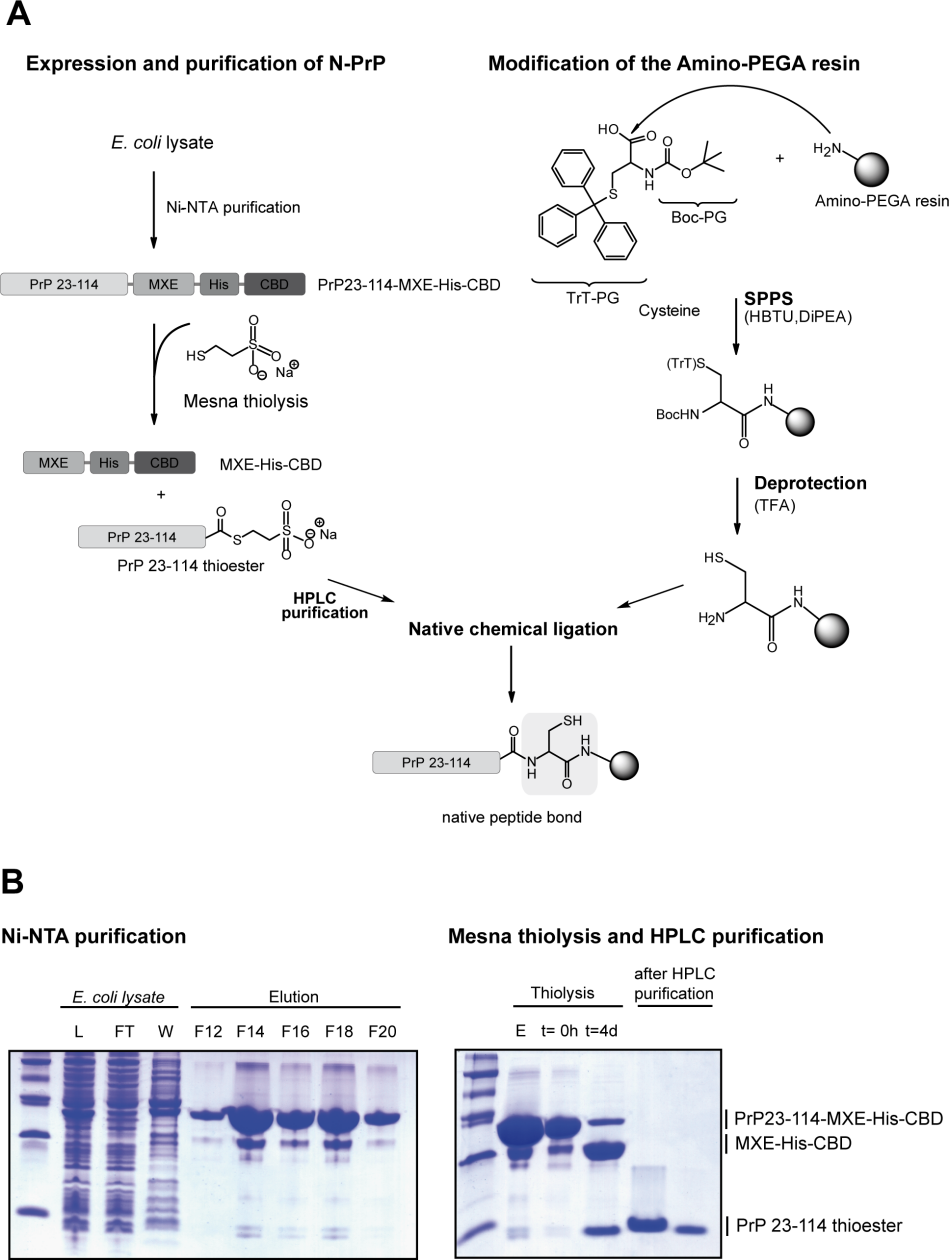
Preparation of PrP23-114 beads. **A. Scheme for generating PrP23-114 beads.** The strategy for the preparation of PrP23-114 coupled to amino-PEGA resin is based on two parallel tiers synthesis. On the left branch, the expressed protein ligation scheme for the synthesis of PrP23-114 thioester is depicted. The right tier illustrates the preparation of the cysteine-modified resin. Finally, the PrP23-114 thioester is coupled to the resin by native chemical ligation. **B. Purification protocol for the preparation of PrP23-114 thioester.** The respective fractions were analyzed by SDS-PAGE and stained with Coomassie Brilliant Blue. Left panel: purification of PrP23-114-MXE-His-CBD. L: *E*. *coli* cell lysate; FT: flow through of proteins not bound to the Ni-NTA column; W: wash; F12–F20: proteins eluted with 250 mM imidazole from the Ni-NTA column. Right panel: mesna thiolysis and HPLC purification. The eluted PrP23-114-MXE-His-CBD (E) was incubated with 300 mM mesna for 4 d (t = 4 d) and purified by HPLC. The pooled fractions of the PrP23-114 thioester were used for native chemical ligation.

To generate a bait for affinity purification of proteins that interact with the IDD of PrP^C^ (N-PrP) we covalently coupled recombinant N-PrP to amino-PEGA resin. The basic idea of this new approach is to couple the protein of interest onto a solid support by using expressed protein ligation methods [36]. The method relies on a cysteine coupled to a PEGA resin and a C-terminal thioester on N-PrP. In the present work a chimeric protein was cloned composed of PrP (aa 23–114) fused to an MXE GyrA intein followed by a His-tag (His) and a chitin-binding domain (CBD). The C-terminally activated thioester, necessary for the ligation step, was generated by cleaving off MXE-His-CBP with 2-mercaptoethanesulfonic acid (mesna). The final binding of PrP (aa 23–114) to the beads was accomplished by chemical ligation [37]. The fusion protein PrP23-114-MXE-His-CBD was expressed in *E*. *coli* and purified by Ni-NTA affinity chromatography (Fig 1A). As illustrated in Fig 1B (left panel, F12-20), PrP23-114-MXE-His-CBD was eluted from the Ni NTA column together with minor amounts of MXE-His-CBD. To cleave N-PrP off the fusion protein and to generate an activated thioester at the C-terminus, the protein was incubated with mesna. After a 4 day incubation in mesna, most of the PrP23-114-MXE-His-CBD fusion protein had been split into MXE-His-CBD and the PrP23-114 thioester. The mixture was fractionated by preparative HPLC using a hydrophobic column to obtain purified PrP23-114 thioester (Fig 1B, right panel). The identity of this sample was confirmed by electrospray ionization mass spectrometry (ESI MS) (data not shown). In parallel, amino-PEGA resin was modified with cysteines by solid phase peptide synthesis (SPPS) [38]. Finally, the C-terminus of the PrP23-114 thioester was covalently coupled to the cysteine-modified PEGA resin via native chemical ligation (Fig 1).

To analyze the human brain interactome of N-PrP and possible alterations linked to Alzheimer’s disease N-PrP beads were incubated in protein extracts prepared from the parietal cortex of an AD- and a non-AD patient to affinity purify N-PrP interactors (Table 1).

**Table 1:** Brain samples used for the analysis.

Sample	Cortex Area	Braak	CERAD	Gender	Age of death	Prnp M129V	ApoE genotype
**AD**	parietal	V	C	male	83	MV	E4/E4
**Non-AD**	parietal	0	0	male	61	VV	E3/E4

CERAD: Consortium to Establish a Registry for Alzheimer’s Disease

**Fig 2:**
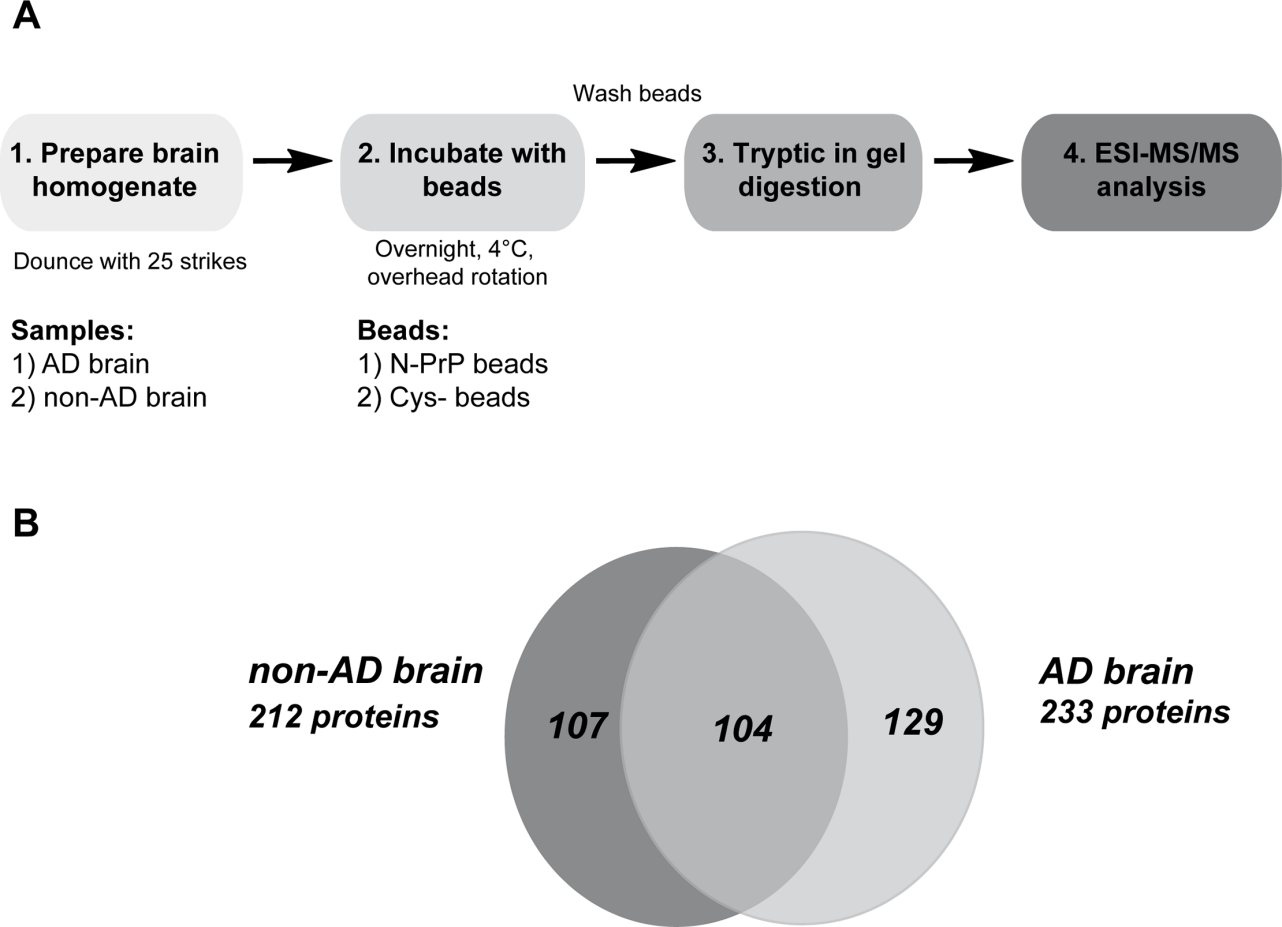
Characterization of N-PrP-interacting proteins. **A. Schematic representation of the experimental approach.** Parietal cortex samples of a non-AD and an AD brain were dissolved in lysis buffer (25% w/v) homogenized with 25 strikes in a glass douncer, and centrifuged (7,500 x g, 4°C). The supernatant was added to the PrP23-114 beads and incubated overnight at 4°C. As a control, lysates were incubated with cysteine-modified beads. To purify interaction partners, beads were washed extensively in lysis buffer. Finally, beads were pelleted by centrifugation and boiled in SDS buffer. The SDS-soluble fraction was separated by SDS-PAGE and proteins co-purified with the beads were identified by ESI-MS/MS after tryptic in-gel digestion. **B. Venn diagram of proteins interacting with PrP23-114 in non-AD and AD brain.** The overlap represents PrP23-114-interacting proteins identified in both brain samples.

As a control, the brain lysates were incubated with beads modified with cysteines but lacking N-PrP. After extensive washing with lysis and washing buffer the beads were pelleted by centrifugation and proteins bound to the beads were eluted by boiling in SDS buffer and identified by electrospray ionization tandem mass spectrometry (nanoESI-MS/MS) (Fig 2).

The analysis was done in triplicates and only those proteins were regarded as specific interactors that were found in all three samples derived from the N-PrP beads and in none of the samples derived from the cysteine-modified beads. In total we identified 233 N-PrP-binding partners in the AD brain (Fig 2, S1 Table) and 212 in the non-AD brain (Fig 2, S2 Table). Among those 104 were overlapping interactors found in both brain homogenates (Fig 2, S3 Table), while 129 proteins were exclusively found in AD brain (Fig 2, S4 Table) and 107 in non-AD brain (Fig 2, S5 Table). By analyzing only one control and one AD sample the conclusion we can draw have certain limitations. Specifically, the total number and identity of the hits might change by analyzing multiple biological replicates.

The specific features of IDDs favor protein-protein interactions. To find out whether N-PrP plays a relevant role in mediating PrP^C^-protein interactions, we searched the literature for previously published PrP-interacting proteins that had been identified using full-length PrP as a bait. Indeed, 19 of the N-PrP-interacting proteins identified in our study had been described previously as PrP interactors (Table 2).

**Table 2:** PrP23-114-interacting proteins identified in the present study that were previously described as interactors of full length PrP.

	Protein	Gene	Reference
1	14-3-3 protein gamma	YWHAG	[39]
2	Amyloid beta	APP	[10]
3	Amyloid beta (A4) precursor protein	APP	[40]
4	ATPase, Na+/K+ transporting, alpha 1 polypeptide	ATP1A1	[40] [39]
5	ATPase, Na+/K+ transporting, beta 1 polypeptide	ATP1B1	[40]
6	Calreticulin	CALR	[39]
7	Calsyntenin-1;Soluble Alc-alpha;CTF1-alpha	CLSTN1	[40]
8	Clathrin, heavy polypeptide	CLTC	[41]
9	Endoplasmin	HSP90B1	[39]
10	Filamin A	FLNA	[42]
11	Growth factor receptor bound protein 2	GRB2	[43]
12	Heat shock protein family A Hsp70	HSPA4L	[44]
13	L1 cell adhesion molecule	L1CAM	[39] [40]
14	Myelin associated glycoprotein	MAG	[40]
15	Neural cell adhesion molecule 1	NCAM1	[40] [39] [45]
16	Stress-induced phosphoprotein 1	STIP1	[46]
17	Synapsin	SYN	[43]
18	Tubulin beta-4A chain	TUB4A	[47]
19	Valosin containing protein	VCP	[41]

These findings support the concept that the intrinsically disordered N-terminal domain is a major mediator of PrP-protein interactions. Apart from the identification of proteins that interact with N-PrP in the context of full-length PrP, our approach is also suited to identify proteins that specifically interact with shedded N-PrP. These interactions seem to be of (patho)physiological interest since mature GPI-anchored PrP^C^ is subjected to proteolytic cleavage at the plasma membrane by a yet unidentified protease at around amino acid 111. Through this processing a soluble N-PrP and a GPI-anchored C-terminal domain is generated [28–31]. Notably, soluble N-PrP is biologically active. For example, it binds to Aβ and thereby abrogates Aβ-induced toxicity [13, 33–35]. In addition, it was reported to activate the G protein-coupled receptor Adgrg6 to promote myelination [32]. Indeed, we were able to reproduce the interaction between N-PrP and Aβ in our assay, but we could not detect the interaction of N-PrP with Adgrg6, since this protein is not expressed in the brain [32].

**Fig 3:**
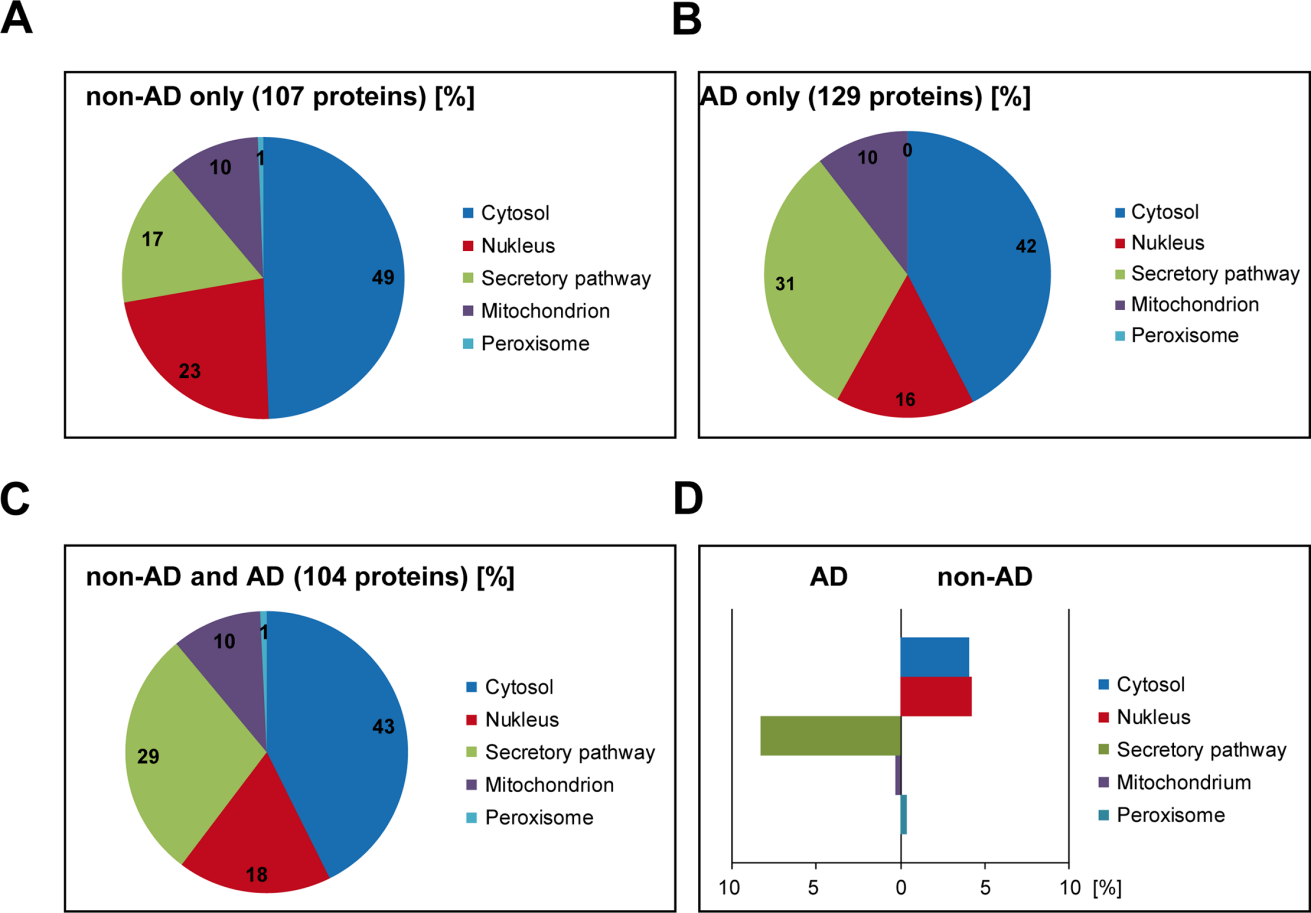
Secretory proteins are enriched in the fraction of PrP23-114-interacting proteins derived from AD brain. A, B, C. Cellular localization of the N-PrP-interactors identified only in non-AD (A) or only in AD brain (B) or identified in both (C). D. Enrichment of N-PrP-interactors in AD or non-AD brain according to their cellular localization.

**Fig 4:**
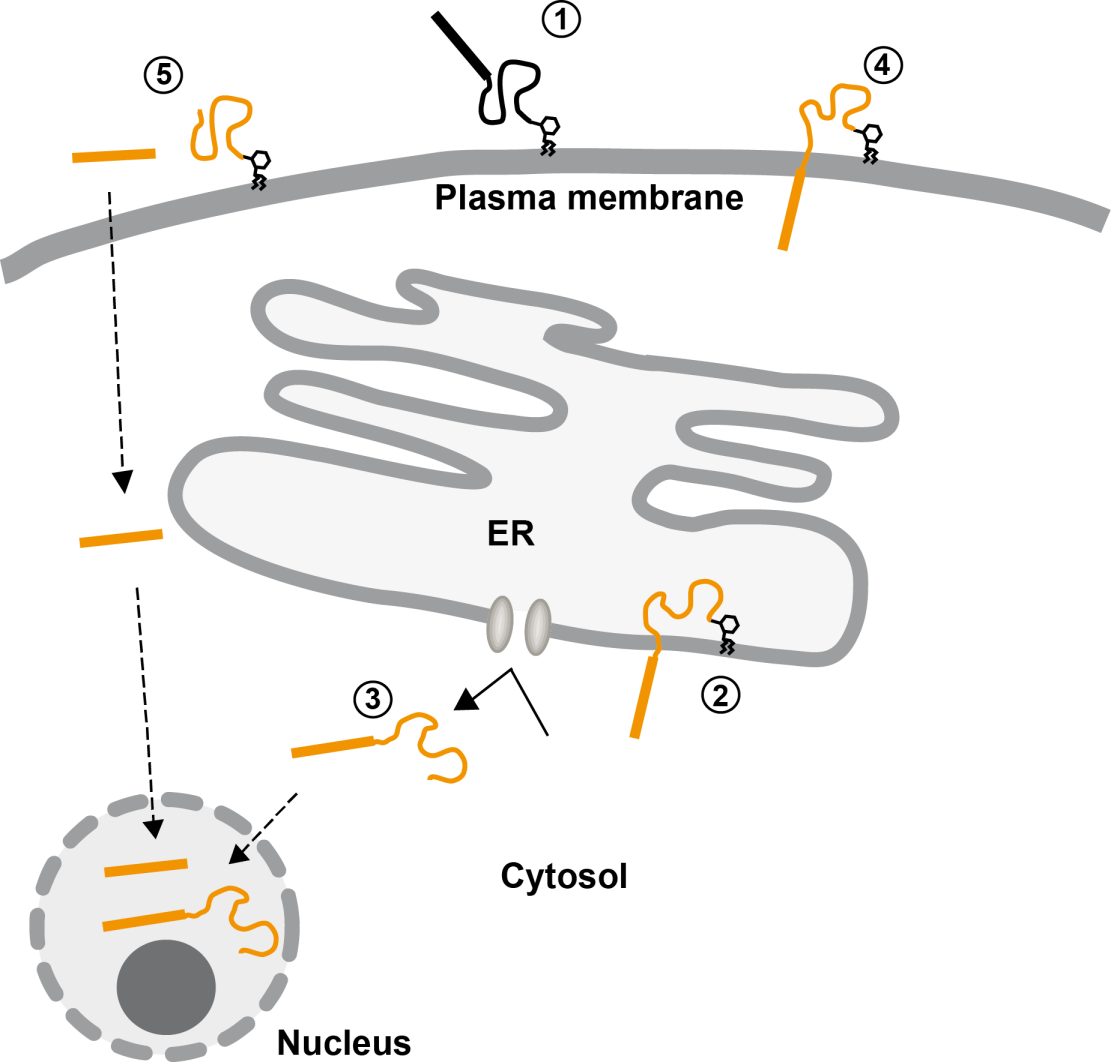
Scheme to illustrate possible cellular localizations of PrP/N-PrP. After maturation in the secretory pathway PrP^C^ is localized at the outer leaflet of the plasma membrane (1). During import into the ER PrP may attain a transmembrane topology, designated PrP^Ctm^, with the N-terminus facing the cytosol (2). After an aborted ER import, or after retrograde transport from the ER lumen full length PrP, or C-terminal truncated variants linked to inherited prion diseases in humans, are missorted to the cytosol and/or nucleus (3). The N-terminal domain of neurotoxic GPI-anchored PrP mutants with a deletion of the hydrophobic domain has been described to penetrate the plasma membrane (4). After proteolytic processing of mature PrP (alpha-cleavage) the polybasic motif can mediate internalization of the liberated N-PrP (5). For references see main text.

In a next step we grouped the identified N-PrP interactors according to their subcellular localization (Fig 3). Unexpectedly, the majority of the identified N-PrP binding partners were not secretory but cytosolic proteins. To interpret these findings, it is important to consider the following findings regarding possible cellular localizations of PrP and/or N-PrP (Fig 4). First, several studies revealed that full-length PrP^C^ can be found in the cytosol or nucleus due to retro-translocation of PrP^C^ from the ER or due to a yet unknown transport process of mature GPI-anchored PrP^C^ from the plasma membrane [48–50]. Second, a topological isoform of PrP with neurotoxic activity, denoted ^Ctm^PrP, can interact with cytosolic proteins via its N-terminal domain facing the cytosol [51, 52]. Third, a class of pathogenic PrP mutants, linked to inherited prion diseases in humans, are inefficiently imported into the ER and are partially localized in the cytosol [53–55]. Fourth, the polybasic motif at the N-terminus of PrP^C^ resembles the trans-activating transcriptional activator (TAT) peptide from human immunodeficiency virus 1 and has cell-penetrating activity [56, 57]. Thus, after proteolytic processing of GPI-anchored PrP^C^ at the plasma membrane the liberated N-PrP can enter the cell and interact with cytosolic/nuclear components.

Another interesting aspect of our study was the identification of two sets of N-PrP-binding partners specific for either the non-AD or AD brain (S4 and S5 Tables). Notably, Aβ was identified as an N-PrP-interactor only in extracts prepared from the AD brain, corroborating previous studies [58, 59]. Since Aβ is also generated under physiological conditions it appears that PrP specifically interacts with a disease associated conformation or species of Aβ. This is supported by the fact that binding of PrP^C^ to pathogenic protein assemblies is determined by their conformation and not their primary sequence [13]. Thus, is seems plausible that at least some interactions of N-PrP are based on conformational differences of the respective proteins in AD versus non-AD samples. Alternatively, the concentrations of the proteins are different due to an altered metabolism in the AD brain.

While the total number of N-PrP-binding proteins was similar in AD and non-AD brain, the fraction of PrP interactors from the secretory pathway was larger in AD brain (31%) compared to non-AD brain (17%) (Fig 3A and 3B). In contrast, a greater number of nuclear proteins was found in the N-PrP interactome in non-AD brain (23% versus 16%) (Fig 3A and 3B). As discussed above, the increased interaction of N-PrP with proteins of the secretory pathway can be explained by conformational differences of the identified proteins due to perturbed ER proteostasis in the diseased brain. Indeed, ER stress seems to play a major role in the occurrence of synaptic dysfunction and neurodegeneration in AD [60].

In conclusion, our study corroborates and extends previously published studies that have successfully identified PrP-interacting proteins. In particular, our findings support the concept that the intrinsically disordered N-terminal domain is a major mediator of PrP interactions. Moreover, by comparing the interactome of N-PrP in extracts prepared from AD and non-AD brain it appears that certain proteins interact with PrP specifically under pathophysiological conditions, possibly due to an altered conformation. It will now be interesting to investigate these interactions in detail to analyze possible functional consequences.

## Materials and methods

### Plasmids/Human tissue/cells

pTXB1 PrP23-114 MXE-His-CBD PrP(aa 23–114) was fused to an MXE GyrA intein short His-tag and a chitin binding domain (CBD) for affinity chromatography and expressed protein ligation. Single amino acid mutation in the Intein allows the creation of an activated C-terminal thioester for native chemical ligation. BL21(DE3) *E*. *coli* cells were used for gene expression.

Human brain tissue: case AD: 244–08, non-AD: 246–08. All autopsies had been performed as clinical autopsies with first-line relatives, next of kin or their legally authorized representatives giving informed consent verbally. Tissue specimens were stored and identified at the Institute of Neuropathology, University Medical Center Hamburg Eppendorf, Germany. The use of specimens obtained at autopsies for research upon anonymization is in accordance with local ethical standards and regulations at the University Medical Center Hamburg-Eppendorf. Furthermore, approval for this study was obtained from the local ethical committee (project number: WF-33/16.) and has been performed in accordance with the ethical standards laid down in an appropriate version of the 1964 Declaration of Helsinki.

### Reagents/Chemicals

Ni-NTA HisTrap FF Columns (5ml) were ordered from GE Healthcare; VYDAC HPLC Column C4-Protein (1/16”) was ordered from GRACE; IPTG and Urea from AppliChem Panreac; NaCl, Tris, Imidazol and mesna from Fluka Analytical; TFA and Acetonitrile (ACN) was ordered from Fischer; Boc-NH-Cys(TrT) from Merck; Amino PEGA resin from EMD Millipore; HBTU, DMF, DiPEA, Gluthathione, Protease Inhibitor Cocktail Mix and Trypsin proteomics grade from Roche ordered at Sigma-Aldrich,

### Expressed protein ligation

*E*. *coli* BL21(DE3) cells transformed with pTXB1 PrP23-144-MXE-His-CBD and selected by ampicillin were grown to an optical density of 0.7. Expression of PrP23-144-MXE-His-CBD was induced by the addition of 1 mM IPTG at 37°C for 3 h. Cells were harvested at 5,000 x g for 20 min and resuspended in cell washing buffer (200 mM NaCl, 50 mM Tris, pH 7.2). Cell membranes were destroyed by sonification (5 x 2 min 20% duty cycle). Membrane debris were pelleted by 16,000 rpm (Ti70, Ultracentrifuge) for 45 min. Supernatant was loaded onto an equilibrated (4 M Urea, 200 mM NaCl, 50 mM Tris, pH 8) Ni-NTA HisTrap FF Colum. Bound PrP23-144-MXE-His-CBD was washed extensively (4 M Urea, 200 mM NaCl, 50 mM Tris, 25 mM Imidazol, pH 8) and eluted from the beads (4 M Urea, 200 mM NaCl, 50 mM Tris, 250 mM Imidazol pH 8). Fractions containing the full-length fusion protein were pooled for dialysis (4 M Urea, 200 mM NaCl, 50 mM Tris, pH 8). Cleavage of the MXE GyrA intein was induced by the addition of 300 mM mesna for 4 d at 4°C. The PrP23-114 thioester was then purified by preparative high performance liquid chromatography (HPLC) using a C4-Protein HPLC column using a gradient (95% A: H_2_O, 0.1% TFA and 5% B: Acetonitrile, 0.08% TFA to 30% A and 70% B in 45 min). Fractions containing PrP23-114 were identified by ESI-MS analysis, pooled and lyophilized.

### Preparation of PrP23-114 -cysteine modified beads

Amino-PEGA resin was modified with a Boc-NH-Cys(TrT) by solid phase peptide synthesis. 0.2 mmol Amino PEGA resin was coupled with 10 eq AA (2 mmol). Amino PEGA resin was swollen in DMF overnight. 2 mmol of Boc-NH-Cys(TrT) were activated by resolving it in 0.5 mol/l HBTU in DMF, 1 ml DiPEA was added. Activated cysteine was added to the resin and incubated for 20 min at room temperature (RT). Coupling procedure was repeated to assure that all amino groups were coupled to the Boc-NH-Cys(TrT). The resin was washed with DMF for 1 min. Coupling efficiency was verified using the Kaiser test which indicates free NH_2_ groups by a simple ninhydrin colour reaction. Deprotection of Boc-NH-Cys(TrT) was achieved by adding 1 ml TFA to the beads which was released immediately. 2 ml of TFA were added and incubated for 1 min at RT. The Cys-Amino PEGA resin was washed extensively for 1 min with DMF and stored dry at 4°C. Cys-Amino PEGA resin was used at the next day for native chemical ligation. Covalent binding of the C-terminal thioester to the cysteine coupled beads was achieved by native chemical ligation. Cys-Amino PEGA was resuspended in 1 ml of ligation buffer (4 M Urea, 200 mM NaCl, 50 mM Tris, 200 mM mesna, pH 8) (c(Cys-Amino PEGA) = 571 mg/ ml). 100 mg of the beads, carring 35 μmol cysteines on their surfaces, were equilibrated to the ligation buffer by centrifugation. Lyophilized PrP23-114 was resolved in ligation buffer (72 mg in 1,2 ml (60 mg/ml)). The molar proportion of PrP: Cys was adjusted to 1:10 (PrP 3,5 μmol:Cys 35 μmol) and incubated for 2 d at RT. Beads were washed at least 10 times with an excess amount of washing buffer (200 mM NaCl, 50 mM Tris, pH 8) by centrifugation, supernatants were discarded. After washing free sulfide groups of the cysteine were blocked by an incubation for 1 h in blocking buffer (50 mM Tris, 60 μM Glutathione, pH 8). Beads were washed extensively and stored at 4°C.

### Preparation of human brain lysates/Interaction assay/Tryptic in-gel digestion

I mg grey matter was homogenized in 3 ml Lysis buffer (150 mM NaCl, 10 mM Tris, 0.5% deoxycholate, 0.5% NP40, pH 7.4), containing a protease inhibitor cocktail Mix. Brain tissue was homogenized with 25 strokes in a glass dounce homogenizer. Homogenates were incubated for 15 min on ice and centrifuged at 7,500 x g at 4°C for 10 min to remove cellular debris. Supernatants were added to equilibrated PrP23-114 beads (200 μl brain lysate/20 mg beads) and incubated over night at 4°C. Beads were washed 3 times with lysis buffer and afterwards 3 times with washing buffer (150 mM NaCl, 10 mM Tris, pH 7.4) to remove NP40 from the beads. Interacting proteins were eluted by boiling in SDS sample buffer. Samples were run on a SDS gel shortly until they reached the separating gel, the gel was coomassie stained and slices containing proteins were cut out of the gel. Tryptic in-gel digestion was performed [61].

### Nano-HPLC/MS/MS

After tryptic digestion and purification, the protein fragments were analyzed by nano-HPLC/MS/MS using an UltimateTM 3000 RSLC nano-HPLC system and a Q ExactiveTM Hybrid Quadrupole-Orbitrap mass spectrometer equipped with a nano-spray source (all Thermo Fisher Scientific). Briefly, the lyophilized tryptic peptides were dissolved in 20 μl 0.1% TFA and 3 μl of these samples were injected and enriched onto a C18 PepMap 100 column (5 μm, 100 Å, 300 μm ID * 5 mm, Dionex, Germany) using 0.1% TFA and a flow rate of 30 μl/min for 5 min. Subsequently, the peptides were separated on a C18 PepMap 100 column (3 μm, 100 Å, 75 μm ID * 25 cm) using a linear gradient starting with 95% solvent A/5% solvent B and increasing to 70.0% solvent A/30.0% solvent B in 90 min with a flow rate of 300 nl/min (solvent A: water containing 0.1% formic acid, solvent B: acetonitril containing 0.1% formic acid). The nano-HPLC was online coupled to the Q Exactive mass spectrometer using a standard coated Pico Tip emitter (ID 20 μm, Tip-ID 10 μM, New Objective, Woburn, MA, USA). Mass range of m/z 300 to 1650 was acquired with a resolution of 70000 for full scan, followed by up to ten high energy collision dissociation (HCD) MS/MS scans of the most intense at least doubly charged ions with a resolution of 17500.

Protein identification and relative quantification were performed using MaxQuant v.1.4.1.1 including the Andromeda search algorithm and searching the human reference proteome of the uniprot dababase. Briefly, a MS/MS ion search was performed for full enzymatic trypsin cleavages allowing two miscleavages. For protein modifications carbamidomethylation was chosen as fixed and oxidation of methionine and acetylation of the N-terminus as variable modifications. The mass accuracy was set to 20 ppm for the first and 6 ppm for the second search. The false discovery rates for peptide and protein identification were set to 0.01. Only proteins for which at least two peptides were identified were chosen for further validation.

To identify possible interaction partners triplicates were further analyzed. Proteins binding to beads without PrP were deleted even if just one unique peptide was found. Only those proteins were counted as a hit when at least two unique peptides were identified and if they were found in each of the three samples (Table AD and non-AD). To identify proteins binding to PrP only in AD brain, proteins identified as a hit in non-AD brain were deleted (vice versa for only non-AD) (Tables only non-AD and only-AD). All experiments were performed in technical triplicates [62].

### Classification according to their cellular localization

To analyze the cellular localization of the N-PrP-interacting partners, proteins were considered individually. The protein database PubMed was used to identify the localization according to the gene symbol and the corresponding organism (Homo sapiens). Localization into the ER, Golgi, vesicles, as well as membrane associated proteins was summarized by the term secretory pathway. Further included in our analysis were nuclear, mitochondrial, cytosolic and peroxisomal localization. Since proteins often show more than one localization each localization was counted and the percentage of the total amount was calculated then.

## Supporting information

S1 Table PrP23-114-interacting proteins in AD brain. (DOCX)

S2 Table PrP23-114-interacting proteins in non-AD brain. (DOCX)

S3 Table PrP23-114-interacting proteins overlapping in AD and non-AD brain. (DOCX)

S4 Table PrP23-114-interacting proteins specific for AD brain. (DOCX)

S5 Table PrP23-114-interacting proteins specific for non-AD brain. (DOCX)
