# Supplementary material for: Alterations in the brain interactome of the intrinsically disordered N-terminal domain of the cellular prion protein (PrPC) in Alzheimer’s disease
Source: PLoS One. 2018 May 23;13(5):e0197659. doi: 10.1371/journal.pone.0197659 (PMC5965872; doi:10.1371/journal.pone.0197659)
Supplement: S2 Table — (DOCX) [file pone.0197659.s002.docx]

S2 Table: PrP23-114-interacting proteins in non-AD brain

|  | **Protein** | **Gene** |
| --- | --- | --- |
| 1 | 28 kDa heat- and acid-stable phosphoprotein | PDAP1 |
| 2 | 2-deoxynucleoside 5-phosphate N-hydrolase 1 | DNPH1 |
| 3 | Activator of 90 kDa heat shock protein ATPase homolog 1 | AHSA1 |
| 4 | Acylamino-acid-releasing enzyme | APEH |
| 5 | Adapter molecule crk | CRK |
| 6 | Adaptin ear-binding coat-associated protein 2 | NECAP2 |
| 7 | ADP-ribosylation factor-like protein 3 | ARL3 |
| 8 | ADP-sugar pyrophosphatase | NUDT5 |
| 9 | Alpha-actinin-4 | ACTN4 |
| 10 | Alpha-ketoglutarate-dependent dioxygenase FTO | FTO |
| 11 | Ankyrin-2 | ANK2 |
| 12 | Annexin A6;Annexin | ANXA6 |
| 13 | Annexin;Annexin A7 | ANXA7 |
| 14 | AP-2 complex subunit alpha-1 | AP2A1 |
| 15 | AP2-associated protein kinase 1 | AAK1 |
| 16 | APC membrane recruitment protein 2 | AMER2 |
| 17 | Aquaporin-4 | AQP4 |
| 18 | Aspartoacylase | ASPA |
| 19 | ATP synthase subunit delta, mitochondrial | ATP5D |
| 20 | Band 4.1-like protein 2 | EPB41L2 |
| 21 | Beta-Ala-His dipeptidase | CNDP1 |
| 22 | Biliverdin reductase A | BLVRA |
| 23 | Branched-chain-amino-acid aminotransferase, cytosolic | BCAT1 |
| 24 | Breast carcinoma-amplified sequence 1 | BCAS1 |
| 25 | Brevican core protein | BCAN |
| 26 | Calcium-regulated heat stable protein 1 | CARHSP1 |
| 27 | Calcyphosin | CAPS |
| 28 | Calreticulin | CALR |
| 29 | Calsyntenin-1;Soluble Alc-alpha;CTF1-alpha | CLSTN1 |
| 30 | cAMP-dependent protein kinase type II-alpha regulatory subunit | PRKAR2A |
| 31 | cAMP-dependent protein kinase type II-beta regulatory subunit | PRKAR2B |
| 32 | CAP-Gly domain-containing linker protein 2 | CLIP2 |
| 33 | Carnosine synthase 1 | CARNS1 |
| 34 | Catechol O-methyltransferase | COMT |
| 35 | CD99 antigen-like protein 2 | CD99L2 |
| 36 | Chloride intracellular channel protein 1 | CLIC1 |
| 37 | Chloride intracellular channel protein 4 | CLIC4 |
| 38 | Clathrin heavy chain 1 | CLTC |
| 39 | Complexin-1 | CPLX1 |
| 40 | Complexin-2 | CPLX2 |
| 41 | COP9 signalosome complex subunit 4 | COPS4 |
| 42 | COP9 signalosome complex subunit 6 | COPS6 |
| 43 | Crk-like protein | CRKL |
| 44 | Cyclin-dependent kinase inhibitor 1B | CDKN1B |
| 45 | Cysteine protease ATG4B | ATG4B |
| 46 | Cytochrome b-c1 complex subunit 1, mitochondrial | UQCRC1 |
| 47 | Cytochrome c oxidase assembly factor 6 homolog | COA6 |
| 48 | Cytochrome c oxidase subunit 5A, mitochondrial | COX5A |
| 49 | Cytosolic 10-formyltetrahydrofolate dehydrogenase | ALDH1L1 |
| 50 | DCC-interacting protein 13-alpha | APPL1 |
| 51 | Dihydrolipoyllysine-residue acetyltransferase component of pyruvate dehydrogenase complex, mitochondrial | DLAT |
| 52 | Disks large homolog 3 | DLG3 |
| 53 | Disks large homolog 4 | DLG4 |
| 54 | DNA fragmentation factor subunit alpha | DFFA |
| 55 | DnaJ homolog subfamily B member 2 | DNAJB2 |
| 56 | Drebrin | DBN1 |
| 57 | Electrogenic sodium bicarbonate cotransporter 1 | SLC4A4 |
| 58 | Elongation factor 1-delta | EEF1D |
| 59 | Elongation factor 2 | EEF2 |
| 60 | Endophilin-A1;Endophilin-A2 | SH3GL2;SH3GL1 |
| 61 | Endoplasmin | HSP90B1 |
| 62 | Ethylmalonyl-CoA decarboxylase | ECHDC1 |
| 63 | Eukaryotic translation initiation factor 4H | EIF4H |
| 64 | Eukaryotic translation initiation factor 5A-1 | EIF5A |
| 65 | Excitatory amino acid transporter 1 | SLC1A3 |
| 66 | Ezrin | EZR |
| 67 | Far upstream element-binding protein 2 | KHSRP |
| 68 | Farnesyl pyrophosphate synthase | FDPS |
| 69 | F-box only protein 2 | FBXO2 |
| 70 | F-box only protein 44 | FBXO44 |
| 71 | Fibulin-1 | FBLN1 |
| 72 | Filamin-A | FLNA |
| 73 | Filamin-C | FLNC |
| 74 | Flavin reductase (NADPH) | BLVRB |
| 75 | Galectin-3-binding protein | LGALS3BP |
| 76 | General vesicular transport factor p115 | USO1 |
| 78 | Gephyrin;Molybdopterin adenylyltransferase;Molybdopterin molybdenumtransferase | GPHN |
| 79 | Glutaredoxin-3 | GLRX3 |
| 80 | Glutathione peroxidase 1 | GPX1 |
| 81 | Glycine--tRNA ligase | GARS |
| 82 | Glycogen phosphorylase, brain form | PYGB |
| 83 | Grancalcin | GCA |
| 84 | Growth factor receptor-bound protein 2 | GRB2 |
| 85 | Haloacid dehalogenase-like hydrolase domain-containing protein 2 | HDHD2 |
| 86 | Haloacid dehalogenase-like hydrolase domain-containing protein 3 | HDHD3 |
| 87 | Hematological and neurological expressed 1 protein | HN1 |
| 88 | Heme-binding protein 2 | HEBP2 |
| 89 | Hepatocyte growth factor-regulated tyrosine kinase substrate | HGS |
| 90 | Hepatoma-derived growth factor | HDGF |
| 91 | Heterogeneous nuclear ribonucleoprotein A3 | HNRNPA3 |
| 92 | Heterogeneous nuclear ribonucleoprotein C-like 1;Heterogeneous nuclear ribonucleoproteins C1/C2 | HNRNPC;HNRNPCL1 |
| 93 | Heterogeneous nuclear ribonucleoproteins A2/B1 | HNRNPA2B1 |
| 94 | High mobility group protein B1;Putative high mobility group protein B1-like 1 | HMGB1;HMGB1P1 |
| 95 | Histidine triad nucleotide-binding protein 1 | HINT1 |
| 96 | Histidine triad nucleotide-binding protein 2, mitochondrial | HINT2 |
| 97 | Histone-lysine N-methyltransferase SETD7 | SETD7 |
| 98 | Homer protein homolog 1 | HOMER1 |
| 99 | Immunity-related GTPase family Q protein | IRGQ |
| 100 | Importin subunit beta-1 | KPNB1 |
| 101 | Importin-5 | IPO5 |
| 102 | Importin-7 | IPO7 |
| 103 | Inorganic pyrophosphatase | PPA1 |
| 104 | Inositol polyphosphate 1-phosphatase | INPP1 |
| 105 | Inositol-tetrakisphosphate 1-kinase | ITPK1 |
| 106 | Iron-sulfur cluster assembly 2 homolog, mitochondrial | ISCA2 |
| 107 | Isocitrate dehydrogenase [NAD] subunit alpha, mitochondrial | IDH3A |
| 108 | JmjC domain-containing protein 7 | JMJD7 |
| 109 | L-aminoadipate-semialdehyde dehydrogenase-phosphopantetheinyl transferase | AASDHPPT |
| 110 | LIM and SH3 domain protein 1 | LASP1 |
| 111 | Lupus La protein | SSB |
| 112 | Microtubule-associated protein 4;Microtubule-associated protein | MAP4 |
| 113 | Microtubule-associated protein RP/EB family member 2 | MAPRE2 |
| 114 | Mitogen-activated protein kinase 1 | MAPK1 |
| 115 | Mitogen-activated protein kinase 3 | MAPK3 |
| 116 | Myelin-associated glycoprotein | MAG |
| 117 | Myosin light chain kinase, smooth muscle;Myosin light chain kinase, smooth muscle, deglutamylated form | MYLK |
| 118 | Myosin light polypeptide 6 | MYL6 |
| 119 | N(G),N(G)-dimethylarginine dimethylaminohydrolase 2 | DDAH2 |
| 120 | N-acetyl-D-glucosamine kinase | NAGK |
| 121 | NAD(P)H-hydrate epimerase | APOA1BP |
| 122 | N-alpha-acetyltransferase 38, NatC auxiliary subunit | NAA38 |
| 123 | NEDD8-activating enzyme E1 catalytic subunit | UBA3 |
| 124 | NEDD8-activating enzyme E1 regulatory subunit | NAE1 |
| 125 | Neudesin | NENF |
| 126 | Neurofilament light polypeptide | NEFL |
| 127 | Neutral alpha-glucosidase AB | GANAB |
| 128 | NSFL1 cofactor p47 | NSFL1C |
| 129 | N-terminal EF-hand calcium-binding protein 1 | NECAB1 |
| 130 | N-terminal EF-hand calcium-binding protein 2 | NECAB2 |
| 131 | Nuclear ubiquitous casein and cyclin-dependent kinase substrate 1 | NUCKS1 |
| 132 | NudC domain-containing protein 2 | NUDCD2 |
| 133 | NudC domain-containing protein 3 | NUDCD3 |
| 134 | Phosphatidylinositol 5-phosphate 4-kinase type-2 alpha;Phosphatidylinositol 5-phosphate 4-kinase type-2 beta | PIP4K2A;PIP4K2B |
| 135 | PITH domain-containing protein 1 | PITHD1 |
| 136 | Platelet-activating factor acetylhydrolase IB subunit gamma | PAFAH1B3 |
| 137 | Poly(ADP-ribose) glycohydrolase ARH3 | ADPRHL2 |
| 138 | Polyadenylate-binding protein 1;Polyadenylate-binding protein 3 | PABPC1;PABPC3 |
| 139 | Programmed cell death protein 5 | PDCD5 |
| 140 | Protein disulfide-isomerase | P4HB |
| 141 | Protein ETHE1, mitochondrial | ETHE1 |
| 142 | Protein farnesyltransferase subunit beta | FNTB;CHURC1-FNTB |
| 143 | Protein farnesyltransferase/geranylgeranyltransferase type-1 subunit alpha | FNTA |
| 144 | Protein LZIC | LZIC |
| 145 | Protein phosphatase 1A | PPM1A |
| 146 | Protein phosphatase 1B | PPM1B |
| 147 | Pterin-4-alpha-carbinolamine dehydratase | PCBD1 |
| 148 | Putative protein phosphatase inhibitor 2-like protein 3;Protein phosphatase inhibitor 2 | PPP1R2P3;PPP1R2 |
| 149 | Ras GTPase-activating-like protein IQGAP1 | IQGAP1 |
| 150 | Ras-related protein Rab-6B | RAB6B |
| 151 | Receptor-type tyrosine-protein phosphatase zeta | PTPRZ1 |
| 152 | Regulator of microtubule dynamics protein 3 | RMDN3 |
| 153 | Reticulon-1 | RTN1 |
| 154 | Retinol-binding protein 1 | RBP1 |
| 155 | Rho-related GTP-binding protein RhoB | RHOB |
| 156 | Ribulose-phosphate 3-epimerase | RPE |
| 157 | Serine/threonine-protein phosphatase 2A 65 kDa regulatory subunit A alpha isoform | PPP2R1A |
| 158 | Serine/threonine-protein phosphatase 2B catalytic subunit alpha isoform;Serine/threonine-protein phosphatase | PPP3CA |
| 159 | Serine-threonine kinase receptor-associated protein | STRAP |
| 160 | Serpin B6 | SERPINB6 |
| 161 | SH3 domain-binding glutamic acid-rich-like protein 3 | SH3BGRL3 |
| 162 | Single-stranded DNA-binding protein, mitochondrial | SSBP1 |
| 163 | Small glutamine-rich tetratricopeptide repeat-containing protein beta | SGTB |
| 164 | Small glutamine-rich tetratricopeptide repeat-containing protein alpha | SGTA |
| 165 | Sorbin and SH3 domain-containing protein 1 | SORBS1 |
| 166 | Spermidine synthase | SRM |
| 167 | Spermine synthase | SMS |
| 168 | Splicing factor U2AF 65 kDa subunit | U2AF2 |
| 169 | Stress-induced-phosphoprotein 1 | STIP1 |
| 170 | Sulfite oxidase, mitochondrial | SUOX |
| 171 | Sulfotransferase 4A1 | SULT4A1 |
| 172 | Synaptopodin | SYNPO |
| 173 | Target of Myb protein 1 | TOM1 |
| 174 | Tax1-binding protein 3 | TAX1BP3 |
| 175 | T-complex protein 1 subunit theta | CCT8 |
| 176 | Tenascin | TNC |
| 177 | Tenascin-R | TNR |
| 178 | Tetratricopeptide repeat protein 1 | TTC1 |
| 179 | Thiamine-triphosphatase | THTPA |
| 180 | Thioredoxin domain-containing protein 17 | TXNDC17 |
| 181 | Thioredoxin, mitochondrial | TXN2 |
| 182 | Thioredoxin-dependent peroxide reductase, mitochondrial | PRDX3 |
| 183 | Thioredoxin-like protein 1 | TXNL1 |
| 184 | THO complex subunit 4 | ALYREF |
| 185 | Tight junction protein ZO-2 | TJP2 |
| 186 | TIP41-like protein | TIPRL |
| 187 | Toll-interacting protein | TOLLIP |
| 188 | TOM1-like protein 2 | TOM1L2 |
| 189 | Transcription elongation factor B polypeptide 1 | TCEB1 |
| 190 | Transcription elongation factor B polypeptide 2 | TCEB2 |
| 191 | Transitional endoplasmic reticulum ATPase | VCP |
| 192 | Translationally-controlled tumor protein | TPT1 |
| 193 | Transport and Golgi organization 2 homolog | TANGO2 |
| 194 | Tripartite motif-containing protein 2 | TRIM2 |
| 195 | Tropomodulin-2 | TMOD2 |
| 196 | Tropomyosin alpha-4 chain | TPM4 |
| 197 | Tryptophan--tRNA ligase, cytoplasmic;T1-TrpRS;T2-TrpRS | WARS |
| 198 | Tubulin alpha-4A chain | TUBA4A |
| 199 | Tubulin-specific chaperone A | TBCA |
| 200 | Tumor protein D54 | TPD52L2 |
| 201 | Ubiquitin-conjugating enzyme E2 Z | UBE2Z |
| 202 | Ubiquitin-like modifier-activating enzyme 1 | UBA1 |
| 203 | UPF0553 protein C9orf64 | C9orf64 |
| 204 | UPF0587 protein C1orf123 | C1orf123 |
| 205 | UPF0696 protein C11orf68 | C11orf68 |
| 206 | Uridine diphosphate glucose pyrophosphatase | NUDT14 |
| 207 | UV excision repair protein RAD23 homolog B | RAD23B |
| 208 | Vimentin | VIM |
| 209 | V-type proton ATPase subunit B, brain isoform | ATP6V1B2 |
| 210 | Xaa-Pro aminopeptidase 1 | XPNPEP1 |
| 211 | X-ray repair cross-complementing protein 5 | XRCC5 |
| 212 | X-ray repair cross-complementing protein 6 | XRCC6 |
