# Supplementary material for: Alterations in the brain interactome of the intrinsically disordered N-terminal domain of the cellular prion protein (PrPC) in Alzheimer’s disease
Source: PLoS One. 2018 May 23;13(5):e0197659. doi: 10.1371/journal.pone.0197659 (PMC5965872; doi:10.1371/journal.pone.0197659)
Supplement: S3 Table — (DOCX) [file pone.0197659.s003.docx]

S3 Table: PrP23-114-interacting proteins overlapping in AD and non-AD brain

|  | **Protein** | **Gene** |
| --- | --- | --- |
| 1 | 28 kDa heat- and acid-stable phosphoprotein | PDAP1 |
| 2 | 2-deoxynucleoside 5-phosphate N-hydrolase 1 | DNPH1 |
| 3 | Acylamino-acid-releasing enzyme | APEH |
| 4 | Adapter molecule crk | CRK |
| 5 | Adaptin ear-binding coat-associated protein 2 | NECAP2 |
| 6 | ADP-sugar pyrophosphatase | NUDT5 |
| 7 | Alpha-actinin-4 | ACTN4 |
| 8 | Alpha-ketoglutarate-dependent dioxygenase FTO | FTO |
| 9 | Ankyrin-2 | ANK2 |
| 10 | Annexin A6;Annexin | ANXA6 |
| 11 | Annexin;Annexin A7 | ANXA7 |
| 12 | AP-2 complex subunit alpha-1 | AP2A1 |
| 13 | AP2-associated protein kinase 1 | AAK1 |
| 14 | APC membrane recruitment protein 2 | AMER2 |
| 15 | Aquaporin-4 | AQP4 |
| 16 | ATP synthase subunit delta, mitochondrial | ATP5D |
| 17 | Band 4.1-like protein 2 | EPB41L2 |
| 18 | Beta-Ala-His dipeptidase | CNDP1 |
| 19 | Biliverdin reductase A | BLVRA |
| 20 | Branched-chain-amino-acid aminotransferase, cytosolic | BCAT1 |
| 21 | Breast carcinoma-amplified sequence 1 | BCAS1 |
| 22 | Brevican core protein | BCAN |
| 23 | Calcium-regulated heat stable protein 1 | CARHSP1 |
| 24 | cAMP-dependent protein kinase type II-alpha regulatory subunit | PRKAR2A |
| 25 | CAP-Gly domain-containing linker protein 2 | CLIP2 |
| 26 | CD99 antigen-like protein 2 | CD99L2 |
| 27 | Chloride intracellular channel protein 1 | CLIC1 |
| 28 | Cytochrome c oxidase subunit 5A, mitochondrial | COX5A |
| 29 | Cytosolic 10-formyltetrahydrofolate dehydrogenase | ALDH1L1 |
| 30 | Dihydrolipoyllysine-residue acetyltransferase component of pyruvate dehydrogenase complex, mitochondrial | DLAT |
| 31 | Disks large homolog 3 | DLG3 |
| 32 | Disks large homolog 4 | DLG4 |
| 33 | DNA fragmentation factor subunit alpha | DFFA |
| 34 | Drebrin | DBN1 |
| 35 | Electrogenic sodium bicarbonate cotransporter 1 | SLC4A4 |
| 36 | Elongation factor 2 | EEF2 |
| 37 | Excitatory amino acid transporter 1 | SLC1A3 |
| 38 | Ezrin | EZR |
| 39 | Farnesyl pyrophosphate synthase | FDPS |
| 40 | Filamin-A | FLNA |
| 41 | Galectin-3-binding protein | LGALS3BP |
| 42 | Gephyrin;Molybdopterin adenylyltransferase;Molybdopterin molybdenumtransferase | GPHN |
| 43 | Glutathione peroxidase 1 | GPX1 |
| 44 | Glycogen phosphorylase, brain form | PYGB |
| 45 | Grancalcin | GCA |
| 46 | Growth factor receptor-bound protein 2 | GRB2 |
| 47 | Haloacid dehalogenase-like hydrolase domain-containing protein 2 | HDHD2 |
| 48 | Haloacid dehalogenase-like hydrolase domain-containing protein 3 | HDHD3 |
| 49 | Heme-binding protein 2 | HEBP2 |
| 50 | Hepatoma-derived growth factor | HDGF |
| 51 | Homer protein homolog 1 | HOMER1 |
| 52 | Inorganic pyrophosphatase | PPA1 |
| 53 | JmjC domain-containing protein 7 | JMJD7 |
| 54 | LIM and SH3 domain protein 1 | LASP1 |
| 55 | Microtubule-associated protein RP/EB family member 2 | MAPRE2 |
| 56 | Mitogen-activated protein kinase 1 | MAPK1 |
| 57 | Myelin-associated glycoprotein | MAG |
| 58 | NAD(P)H-hydrate epimerase | APOA1BP |
| 59 | Neudesin | NENF |
| 60 | NSFL1 cofactor p47 | NSFL1C |
| 61 | N-terminal EF-hand calcium-binding protein 1 | NECAB1 |
| 62 | N-terminal EF-hand calcium-binding protein 2 | NECAB2 |
| 63 | Nuclear ubiquitous casein and cyclin-dependent kinase substrate 1 | NUCKS1 |
| 64 | PITH domain-containing protein 1 | PITHD1 |
| 65 | Polyadenylate-binding protein 1;Polyadenylate-binding protein 3 | PABPC1;PABPC3 |
| 66 | Protein disulfide-isomerase | P4HB |
| 67 | Protein phosphatase 1A | PPM1A |
| 68 | Pterin-4-alpha-carbinolamine dehydratase | PCBD1 |
| 69 | Ras GTPase-activating-like protein IQGAP1 | IQGAP1 |
| 70 | Receptor-type tyrosine-protein phosphatase zeta | PTPRZ1 |
| 71 | Regulator of microtubule dynamics protein 3 | RMDN3 |
| 72 | Reticulon-1 | RTN1 |
| 73 | Retinol-binding protein 1 | RBP1 |
| 74 | Rho-related GTP-binding protein RhoB | RHOB |
| 75 | Serine/threonine-protein phosphatase 2B catalytic subunit alpha isoform;Serine/threonine-protein phosphatase | PPP3CA |
| 76 | Serpin B6 | SERPINB6 |
| 77 | Small glutamine-rich tetratricopeptide repeat-containing protein beta | SGTB |
| 78 | Small glutamine-rich tetratricopeptide repeat-containing protein alpha | SGTA |
| 79 | Sorbin and SH3 domain-containing protein 1 | SORBS1 |
| 80 | Spermidine synthase | SRM |
| 81 | Splicing factor U2AF 65 kDa subunit | U2AF2 |
| 82 | Stress-induced-phosphoprotein 1 | STIP1 |
| 83 | Sulfite oxidase, mitochondrial | SUOX |
| 84 | Tenascin | TNC |
| 85 | Tenascin-R | TNR |
| 86 | Tetratricopeptide repeat protein 1 | TTC1 |
| 87 | Thiamine-triphosphatase | THTPA |
| 88 | Thioredoxin domain-containing protein 17 | TXNDC17 |
| 89 | Thioredoxin-dependent peroxide reductase, mitochondrial | PRDX3 |
| 90 | Thioredoxin-like protein 1 | TXNL1 |
| 91 | TIP41-like protein | TIPRL |
| 92 | Toll-interacting protein | TOLLIP |
| 93 | TOM1-like protein 2 | TOM1L2 |
| 94 | Translationally-controlled tumor protein | TPT1 |
| 95 | Transport and Golgi organization 2 homolog | TANGO2 |
| 96 | Tropomyosin alpha-4 chain | TPM4 |
| 97 | Tubulin-specific chaperone A | TBCA |
| 98 | Tumor protein D54 | TPD52L2 |
| 99 | Ubiquitin-conjugating enzyme E2 Z | UBE2Z |
| 100 | UPF0553 protein C9orf64 | C9orf64 |
| 101 | UPF0696 protein C11orf68 | C11orf68 |
| 102 | Uridine diphosphate glucose pyrophosphatase | NUDT14 |
| 103 | V-type proton ATPase subunit B, brain isoform | ATP6V1B2 |
| 104 | Xaa-Pro aminopeptidase 1 | XPNPEP1 |
