# Supplementary material for: Alterations in the brain interactome of the intrinsically disordered N-terminal domain of the cellular prion protein (PrPC) in Alzheimer’s disease
Source: PLoS One. 2018 May 23;13(5):e0197659. doi: 10.1371/journal.pone.0197659 (PMC5965872; doi:10.1371/journal.pone.0197659)
Supplement: S4 Table — (DOCX) [file pone.0197659.s004.docx]

S4 Table: PrP23-114-interacting proteins specific for AD brain

|  | **Protein** | **Gene** |
| --- | --- | --- |
| 1 | 1-phosphatidylinositol 4,5-bisphosphate phosphodiesterase beta-1 | PLCB1 |
| 2 | Adenosylhomocysteinase | AHCY |
| 3 | Adenosylhomocysteinase;Putative adenosylhomocysteinase 2;Putative adenosylhomocysteinase 3 | AHCYL2;AHCYL1 |
| 4 | Afadin | MLLT4 |
| 5 | Alpha-actinin-1 | ACTN1 |
| 6 | Arf-GAP with GTPase, ANK repeat and PH domain-containing protein 3 | AGAP3 |
| 7 | ATP synthase subunit alpha, mitochondrial;ATP synthase subunit alpha | ATP5A1 |
| 8 | Band 4.1-like protein 1 | EPB41L1 |
| 9 | Calcium/calmodulin-dependent protein kinase type II subunit alpha | CAMK2A |
| 10 | Calcium/calmodulin-dependent protein kinase type II subunit beta | CAMK2B |
| 11 | Calcium/calmodulin-dependent protein kinase type II subunit delta | CAMK2D |
| 12 | Calcium/calmodulin-dependent protein kinase type II subunit gamma | CAMK2G |
| 13 | Calpastatin | CAST |
| 14 | Carboxypeptidase E | CPE |
| 15 | Cellular retinoic acid-binding protein 1 | CRABP1 |
| 16 | Citron Rho-interacting kinase | CIT |
| 17 | Clathrin light chain B | CLTB |
| 18 | Cleavage stimulation factor subunit 2 | CSTF2 |
| 19 | Complement C3;Complement C3 beta chain;Complement C3 alpha chain;C3a anaphylatoxin;Acylation stimulating protein;Complement C3b alpha chain;Complement C3c alpha chain fragment 1;Complement C3dg fragment;Complement C3g fragment;Complement C3d fragment;Complement C3f fragment;Complement C3c alpha chain fragment 2 | C3 |
| 20 | Copine-3;Copine-2;Copine-9;Copine-4;Copine-6;Copine-8;Copine-5;Copine-7 | CPNE3;CPNE8;CPNE5;CPNE2;CPNE9;CPNE4;CPNE6;CPNE7 |
| 21 | Cytochrome c oxidase subunit 6B1 | COX6B1 |
| 22 | Delta-aminolevulinic acid dehydratase | ALAD |
| 23 | Density-regulated protein | DENR |
| 24 | Deoxyuridine 5-triphosphate nucleotidohydrolase, mitochondrial | DUT |
| 25 | Dihydrolipoyllysine-residue succinyltransferase component of 2-oxoglutarate dehydrogenase complex, mitochondrial | DLST |
| 26 | Dihydropyrimidinase-related protein 5 | DPYSL5 |
| 27 | Disks large homolog 1 | DLG1 |
| 28 | Disks large homolog 2 | DLG2 |
| 29 | DNA damage-binding protein 1 | DDB1 |
| 30 | DnaJ homolog subfamily A member 2 | DNAJA2 |
| 31 | Epidermal growth factor receptor substrate 15-like 1 | EPS15L1 |
| 32 | Eukaryotic initiation factor 4A-II | EIF4A2 |
| 33 | Eukaryotic translation initiation factor 4B | EIF4B |
| 34 | F-actin-capping protein subunit alpha-1 | CAPZA1 |
| 35 | F-actin-capping protein subunit beta | CAPZB |
| 36 | Far upstream element-binding protein 1 | FUBP1 |
| 37 | Fatty acid-binding protein, brain | FABP7;DKFZp547J2313 |
| 38 | Fibrinogen alpha chain;Fibrinopeptide A;Fibrinogen alpha chain | FGA |
| 39 | Frataxin, mitochondrial;Frataxin intermediate form;Frataxin(56-210);Frataxin(78-210);Frataxin mature form | FXN |
| 40 | Gamma-aminobutyric acid receptor-associated protein-like 1;Gamma-aminobutyric acid receptor-associated protein | GABARAPL1;GABARAP |
| 41 | Gap junction alpha-1 protein | GJA1 |
| 42 | Glutamate dehydrogenase 1, mitochondrial;Glutamate dehydrogenase;Glutamate dehydrogenase 2, mitochondrial | GLUD1;GLUD2 |
| 43 | Glycogen debranching enzyme;4-alpha-glucanotransferase;Amylo-alpha-1,6-glucosidase | AGL |
| 44 | GTPase HRas;GTPase HRas, N-terminally processed | HRAS |
| 45 | HD domain-containing protein 2 | HDDC2 |
| 46 | Heat shock 70 kDa protein 4L | HSPA4L |
| 47 | Heterogeneous nuclear ribonucleoprotein H3 | HNRNPH3 |
| 48 | Hsp70-binding protein 1 | HSPBP1 |
| 49 | Hyaluronan and proteoglycan link protein 1 | HAPLN1 |
| 50 | Inorganic pyrophosphatase 2, mitochondrial | PPA2 |
| 51 | Inositol-3-phosphate synthase 1 | ISYNA1 |
| 52 | Isoamyl acetate-hydrolyzing esterase 1 homolog | IAH1 |
| 53 | Isoaspartyl peptidase/L-asparaginase;Isoaspartyl peptidase/L-asparaginase alpha chain;Isoaspartyl peptidase/L-asparaginase beta chain | ASRGL1 |
| 54 | Lamin-B2 | LMNB2 |
| 55 | Laminin subunit beta-2 | LAMB2 |
| 56 | Latexin | LXN |
| 57 | Matrin-3 | MATR3 |
| 58 | Membrane-associated guanylate kinase, WW and PDZ domain-containing protein 2 | MAGI2 |
| 59 | Metallo-beta-lactamase domain-containing protein 1 | MBLAC1 |
| 60 | Methionine adenosyltransferase 2 subunit beta | MAT2B |
| 61 | Methionine-R-sulfoxide reductase B2, mitochondrial | MSRB2 |
| 62 | Microtubule-actin cross-linking factor 1, isoforms 1/2/3/5 | MACF1 |
| 63 | NADH dehydrogenase [ubiquinone] iron-sulfur protein 6, mitochondrial | NDUFS6 |
| 64 | NADH-cytochrome b5 reductase 3;NADH-cytochrome b5 reductase 3 membrane-bound form;NADH-cytochrome b5 reductase 3 soluble form | CYB5R3 |
| 65 | NEDD8 | NEDD8;NEDD8-MDP1 |
| 66 | Neural cell adhesion molecule 1 | NCAM1 |
| 67 | Neural cell adhesion molecule L1 | L1CAM |
| 68 | Neuronal membrane glycoprotein M6-a | GPM6A |
| 69 | Neuronal pentraxin-1 | NPTX1 |
| 70 | Neuroserpin | SERPINI1 |
| 71 | NIF3-like protein 1 | NIF3L1 |
| 72 | Omega-amidase NIT2 | NIT2 |
| 73 | Peptidyl-prolyl cis-trans isomerase FKBP4;Peptidyl-prolyl cis-trans isomerase FKBP4, N-terminally processed;Peptidyl-prolyl cis-trans isomerase | FKBP4 |
| 74 | Phosphoacetylglucosamine mutase | PGM3 |
| 75 | Phosphoribosylformylglycinamidine synthase | PFAS |
| 76 | Plasma membrane calcium-transporting ATPase 1 | PMCA1 |
| 77 | Polyadenylate-binding protein-interacting protein 1 | PAIP1 |
| 78 | Prostaglandin reductase 2 | PTGR2 |
| 79 | Proteasome subunit alpha type;Proteasome subunit alpha type-6 | PSMA6 |
| 80 | Proteasome subunit alpha type-5 | PSMA5 |
| 81 | Proteasome subunit alpha type-7;Proteasome subunit alpha type-7-like | PSMA7;PSMA8 |
| 82 | Protein bassoon | BSN |
| 83 | Protein kinase C and casein kinase substrate in neurons protein 2 | PACSIN2 |
| 84 | Protein NDRG4 | NDRG4 |
| 85 | Protein TFG | TFG |
| 86 | Pyridoxal phosphate phosphatase | PDXP |
| 87 | Quinone oxidoreductase-like protein 1 | CRYZL1 |
| 88 | Rab-like protein 6 | RABL6 |
| 89 | Rabphilin-3A | RPH3A |
| 90 | Ras-related protein Rab-27B;Ras-related protein Rab-27A | RAB27B;RAB27A |
| 91 | Ras-related protein Ral-A | RALA |
| 92 | Ribosyldihydronicotinamide dehydrogenase [quinone] | NQO2 |
| 93 | S-adenosylmethionine synthase isoform type-2;S-adenosylmethionine synthase | MAT2A |
| 94 | Secretogranin-1;GAWK peptide;CCB peptide | CHGB |
| 95 | Secretory carrier-associated membrane protein 1 | SCAMP1 |
| 96 | Serine/threonine-protein phosphatase 2B catalytic subunit beta isoform;Serine/threonine-protein phosphatase | PPP3CB |
| 97 | Serpin B9 | SERPINB9 |
| 98 | SH3 domain-binding glutamic acid-rich-like protein 2 | SH3BGRL2 |
| 99 | SH3 domain-containing kinase-binding protein 1 | SH3KBP1 |
| 100 | SH3-containing GRB2-like protein 3-interacting protein 1 | SGIP1 |
| 101 | Small nuclear ribonucleoprotein Sm D1 | SNRPD1 |
| 102 | S-methyl-5-thioadenosine phosphorylase;Purine nucleoside phosphorylase | MTAP |
| 103 | Sodium/potassium-transporting ATPase subunit alpha-1 | ATP1A1 |
| 104 | Sodium/potassium-transporting ATPase subunit alpha-2 | ATP1A2 |
| 105 | Sodium/potassium-transporting ATPase subunit alpha-3 | ATP1A3 |
| 106 | Sodium/potassium-transporting ATPase subunit beta-1 | ATP1B1 |
| 107 | Sodium-driven chloride bicarbonate exchanger | SLC4A10 |
| 108 | Src substrate cortactin | CTTN |
| 109 | Succinate-semialdehyde dehydrogenase, mitochondrial | ALDH5A1 |
| 110 | Synapsin-2 | SYN2 |
| 111 | Synaptojanin-2-binding protein | SYNJ2BP |
| 112 | Synaptotagmin-1 | SYT1 |
| 113 | Syntaxin-12 | STX12 |
| 114 | Talin-1 | TLN1 |
| 115 | Talin-2 | TLN2 |
| 116 | TBC1 domain family member 24 | TBC1D24 |
| 117 | Thioredoxin domain-containing protein 12 | TXNDC12 |
| 118 | Transformer-2 protein homolog beta | TRA2B |
| 119 | Transforming protein RhoA;Rho-related GTP-binding protein RhoC | RHOA;RHOC |
| 120 | Translin | TSN |
| 121 | Transthyretin | TTR |
| 122 | Tripeptidyl-peptidase 1 | TPP1 |
| 123 | Tumor protein D52 | TPD52 |
| 124 | Twinfilin-2 | TWF2 |
| 125 | Tyrosine-protein phosphatase non-receptor type 11 | PTPN11 |
| 126 | U1 small nuclear ribonucleoprotein A;U2 small nuclear ribonucleoprotein B | SNRPA;SNRPB2 |
| 127 | Vesicle-associated membrane protein-associated protein A | VAPA |
| 128 | V-type proton ATPase catalytic subunit A | ATP6V1A |
| 129 | WD repeat-containing protein 91 | WDR91 |
