# Supplementary material for: Alterations in the brain interactome of the intrinsically disordered N-terminal domain of the cellular prion protein (PrPC) in Alzheimer’s disease
Source: PLoS One. 2018 May 23;13(5):e0197659. doi: 10.1371/journal.pone.0197659 (PMC5965872; doi:10.1371/journal.pone.0197659)
Supplement: S5 Table — (DOCX) [file pone.0197659.s005.docx]

S5 Table: PrP23-114-interacting proteins specific for non-AD brain

|  | **Protein** | **Gene** |
| --- | --- | --- |
| 1 | Transcription elongation factor B polypeptide 1 | TCEB1 |
| 2 | Platelet-activating factor acetylhydrolase IB subunit gamma | PAFAH1B3 |
| 3 | Histidine triad nucleotide-binding protein 2, mitochondrial | HINT2 |
| 4 | Ethylmalonyl-CoA decarboxylase | ECHDC1 |
| 5 | Putative protein phosphatase inhibitor 2-like protein 3;Protein phosphatase inhibitor 2 | PPP1R2P3;PPP1R2 |
| 6 | Protein phosphatase 1B | PPM1B |
| 7 | X-ray repair cross-complementing protein 5 | XRCC5 |
| 8 | F-box only protein 44 | FBXO44 |
| 9 | Iron-sulfur cluster assembly 2 homolog, mitochondrial | ISCA2 |
| 10 | Far upstream element-binding protein 2 | KHSRP |
| 11 | Heterogeneous nuclear ribonucleoprotein A3 | HNRNPA3 |
| 12 | Single-stranded DNA-binding protein, mitochondrial | SSBP1 |
| 13 | Cyclin-dependent kinase inhibitor 1B | CDKN1B |
| 14 | Glutaredoxin-3 | GLRX3 |
| 15 | Calcyphosin | CAPS |
| 16 | Heterogeneous nuclear ribonucleoproteins A2/B1 | HNRNPA2B1 |
| 17 | Tryptophan--tRNA ligase, cytoplasmic;T1-TrpRS;T2-TrpRS | WARS |
| 18 | UV excision repair protein RAD23 homolog B | RAD23B |
| 19 | Lupus La protein | SSB |
| 20 | Protein LZIC | LZIC |
| 21 | Inositol-tetrakisphosphate 1-kinase | ITPK1 |
| 22 | L-aminoadipate-semialdehyde dehydrogenase-phosphopantetheinyl transferase | AASDHPPT |
| 23 | Calreticulin | CALR |
| 24 | T-complex protein 1 subunit theta | CCT8 |
| 25 | Activator of 90 kDa heat shock protein ATPase homolog 1 | AHSA1 |
| 26 | Histone-lysine N-methyltransferase SETD7 | SETD7 |
| 27 | X-ray repair cross-complementing protein 6 | XRCC6 |
| 28 | Protein farnesyltransferase subunit beta | FNTB;CHURC1-FNTB |
| 29 | Sulfotransferase 4A1 | SULT4A1 |
| 30 | Tubulin alpha-4A chain | TUBA4A |
| 31 | Transcription elongation factor B polypeptide 2 | TCEB2 |
| 32 | Filamin-C | FLNC |
| 33 | N-alpha-acetyltransferase 38, NatC auxiliary subunit | NAA38 |
| 34 | Microtubule-associated protein 4;Microtubule-associated protein | MAP4 |
| 35 | NudC domain-containing protein 2 | NUDCD2 |
| 36 | Phosphatidylinositol 5-phosphate 4-kinase type-2 alpha;Phosphatidylinositol 5-phosphate 4-kinase type-2 beta | PIP4K2A;PIP4K2B |
| 37 | Mitogen-activated protein kinase 3 | MAPK3 |
| 38 | Cysteine protease ATG4B | ATG4B |
| 39 | Spermine synthase | SMS |
| 40 | N-acetyl-D-glucosamine kinase | NAGK |
| 41 | Eukaryotic translation initiation factor 5A-1 | EIF5A |
| 42 | DCC-interacting protein 13-alpha | APPL1 |
| 43 | Importin-7 | IPO7 |
| 44 | ADP-ribosylation factor-like protein 3 | ARL3 |
| 45 | Eukaryotic translation initiation factor 4H | EIF4H |
| 46 | Catechol O-methyltransferase | COMT |
| 47 | Serine-threonine kinase receptor-associated protein | STRAP |
| 48 | Chloride intracellular channel protein 4 | CLIC4 |
| 49 | Carnosine synthase 1 | CARNS1 |
| 50 | F-box only protein 2 | FBXO2 |
| 51 | Endoplasmin | HSP90B1 |
| 52 | Neutral alpha-glucosidase AB | GANAB |
| 53 | Ras-related protein Rab-6B | RAB6B |
| 54 | Ribulose-phosphate 3-epimerase | RPE |
| 55 | COP9 signalosome complex subunit 6 | COPS6 |
| 56 | Histidine triad nucleotide-binding protein 1 | HINT1 |
| 57 | Target of Myb protein 1 | TOM1 |
| 58 | UPF0587 protein C1orf123 | C1orf123 |
| 59 | Protein farnesyltransferase/geranylgeranyltransferase type-1 subunit alpha | FNTA |
| 60 | Serine/threonine-protein phosphatase 2A 65 kDa regulatory subunit A alpha isoform | PPP2R1A |
| 61 | Immunity-related GTPase family Q protein | IRGQ |
| 62 | NEDD8-activating enzyme E1 catalytic subunit | UBA3 |
| 63 | Glycine--tRNA ligase | GARS |
| 64 | Importin subunit beta-1 | KPNB1 |
| 65 | Cytochrome b-c1 complex subunit 1, mitochondrial | UQCRC1 |
| 66 | Fibulin-1 | FBLN1 |
| 67 | Programmed cell death protein 5 | PDCD5 |
| 68 | Ubiquitin-like modifier-activating enzyme 1 | UBA1 |
| 69 | High mobility group protein B1;Putative high mobility group protein B1-like 1 | HMGB1;HMGB1P1 |
| 70 | Myosin light polypeptide 6 | MYL6 |
| 71 | Neurofilament light polypeptide | NEFL |
| 72 | Tax1-binding protein 3 | TAX1BP3 |
| 73 | Synaptopodin | SYNPO |
| 74 | Thioredoxin, mitochondrial | TXN2 |
| 75 | Tropomodulin-2 | TMOD2 |
| 76 | Heterogeneous nuclear ribonucleoprotein C-like 1;Heterogeneous nuclear ribonucleoproteins C1/C2 | HNRNPC;HNRNPCL1 |
| 77 | Tight junction protein ZO-2 | TJP2 |
| 78 | COP9 signalosome complex subunit 4 | COPS4 |
| 79 | NEDD8-activating enzyme E1 regulatory subunit | NAE1 |
| 80 | Tripartite motif-containing protein 2 | TRIM2 |
| 81 | Hepatocyte growth factor-regulated tyrosine kinase substrate | HGS |
| 82 | General vesicular transport factor p115 | USO1 |
| 83 | Endophilin-A1;Endophilin-A2 | SH3GL2;SH3GL1 |
| 84 | Elongation factor 1-delta | EEF1D |
| 85 | Inositol polyphosphate 1-phosphatase | INPP1 |
| 86 | Myosin light chain kinase, smooth muscle;Myosin light chain kinase, smooth muscle, deglutamylated form | MYLK |
| 87 | Vimentin | VIM |
| 88 | Aspartoacylase | ASPA |
| 89 | N(G),N(G)-dimethylarginine dimethylaminohydrolase 2 | DDAH2 |
| 90 | THO complex subunit 4 | ALYREF |
| 91 | Calsyntenin-1;Soluble Alc-alpha;CTF1-alpha | CLSTN1 |
| 92 | Complexin-1 | CPLX1 |
| 93 | cAMP-dependent protein kinase type II-beta regulatory subunit | PRKAR2B |
| 94 | Protein ETHE1, mitochondrial | ETHE1 |
| 95 | Cytochrome c oxidase assembly factor 6 homolog | COA6 |
| 96 | NudC domain-containing protein 3 | NUDCD3 |
| 97 | Isocitrate dehydrogenase [NAD] subunit alpha, mitochondrial | IDH3A |
| 98 | Flavin reductase (NADPH) | BLVRB |
| 99 | Complexin-2 | CPLX2 |
| 100 | Transitional endoplasmic reticulum ATPase | VCP |
| 101 | Importin-5 | IPO5 |
| 102 | Hematological and neurological expressed 1 protein | HN1 |
| 103 | DnaJ homolog subfamily B member 2 | DNAJB2 |
| 104 | Crk-like protein | CRKL |
| 105 | Poly(ADP-ribose) glycohydrolase ARH3 | ADPRHL2 |
| 106 | Clathrin heavy chain 1 | CLTC |
| 107 | SH3 domain-binding glutamic acid-rich-like protein 3 | SH3BGRL3 |
